# T-Lymphocytes Traffic into the Brain across the Blood-CSF Barrier: Evidence Using a Reconstituted Choroid Plexus Epithelium

**DOI:** 10.1371/journal.pone.0150945

**Published:** 2016-03-04

**Authors:** Nathalie Strazielle, Rita Creidy, Christophe Malcus, José Boucraut, Jean-François Ghersi-Egea

**Affiliations:** 1 Brain-i, Lyon, France; 2 Blood-brain interfaces exploratory platform BIP and FLUID Team, Lyon Neurosciences Research Center, INSERM U1028 CRNS UMR 5292, Université Claude Bernard Lyon-1, Lyon, France; 3 CRN2M, CNRS UMR 7286, Aix Marseille Univ, 13344, Marseille Cedex 15, France; 4 Service d’Immunologie, Hôpital Edouard Herriot, Hospices Civils de Lyon, Lyon, France; 5 Plateforme de Biologie médicale, UF d'Immunologie, Centre Hospitalier Intercommunal de Créteil, Créteil, France; Hungarian Academy of Sciences, HUNGARY

## Abstract

An emerging concept of normal brain immune surveillance proposes that recently and moderately activated central memory T lymphocytes enter the central nervous system (CNS) directly into the cerebrospinal fluid (CSF) via the choroid plexus. Within the CSF space, T cells inspect the CNS environment for cognate antigens. This gate of entry into the CNS could also prevail at the initial stage of neuroinflammatory processes. To actually demonstrate T cell migration across the choroidal epithelium forming the blood-CSF barrier, an in vitro model of the rat blood-CSF barrier was established in an “inverse” configuration that enables cell transmigration studies in the basolateral to apical, i.e. blood/stroma to CSF direction. Structural barrier features were evaluated by immunocytochemical analysis of tight junction proteins, functional barrier properties were assessed by measuring the monolayer permeability to sucrose and the active efflux transport of organic anions. The migratory behaviour of activated T cells across the choroidal epithelium was analysed in the presence and absence of chemokines. The migration pathway was examined by confocal microscopy. The inverse rat BCSFB model reproduces the continuous distribution of tight junction proteins at cell margins, the restricted paracellular permeability, and polarized active transport mechanisms, which all contribute to the barrier phenotype in vivo. Using this model, we present experimental evidence of T cell migration across the choroidal epithelium. Cell migration appears to occur via a paracellular route without disrupting the restrictive barrier properties of the epithelial interface. Apical chemokine addition strongly stimulates T cell migration across the choroidal epithelium. The present data provide evidence for the controlled migration of T cells across the blood-CSF barrier into brain. They further indicate that this recruitment route is sensitive to CSF-borne chemokines, extending the relevance of this migration pathway to neuroinflammatory and neuroinfectious disorders which are typified by elevated chemokine levels in CSF.

## Introduction

The cerebrospinal fluid (CSF) is recognized as a predominant route of T-cell trafficking within the central nervous system (CNS). It is considered as the only site in the healthy brain that contains CD4+ T cells [1,2]. These cells are primarily central memory and effector memory cells and express high levels of the adhesion molecule P-selectin glycoprotein ligand 1 (PSGL-1) [1,3,4,5]. The involvement in neuroimmune surveillance of P-selectin, a major counterligand for PSGL-1 [6] responsible for the initial tethering and rolling of leucocytes on blood vessels, was highlighted by Carrithers and collaborators [7]. They reported that P- selectin facilitates the early migration of activated PSGL-1+ splenocytes and CD4 T_H_1 cells in the healthy mouse brain. In the non-inflamed brain in which the resting microvessel endothelium forming the blood-brain barrier does not support cell extravasation [1,3,8], P-selectin is confined to the choroid plexus and the meningeal vessels as shown in mouse and human [4,7], indicating that leucocytes can in theory access CSF at both levels of the fluid flowing pathway. They can enter upstream via the choroid plexus into the ventricular spaces from where they follow the flow, or they can extravasate downstream, from subpial vessels into the subarachnoid spaces.

A number of factual observations support the former route through the choroid plexus, during normal immunosurveillance and in the early phase of neuroinflammatory processes. Analysis of matched ventricular and lumbar CSF samples from patients with normal pressure hydrocephalus showed identical number of leucocytes per volume unit, and identical leucocyte differential counts [5]. The paired CSF samples also displayed similar proportions of T-cell subsets, with a majority of CD4+ T cells. In accord with their transchoroidal route of migration, T cells are present in the choroid plexus stroma. They have been detected in human and murine tissue [4,9] and their number increased to some extent after non-specific peripheral immune activation [9,10]. It was then shown that initiation of experimental autoimmune encephalomyelitis requires brain entry of T_H_17 cells though the choroid plexus. Their penetration in the CNS is dependent on the chemokine receptor CCR6, whose chemokine ligand CCL20 is constitutively synthesized by the human, murine, and rodent choroidal epithelium ([11], and unpublished results). This choroidal pathway may also be relevant for pathogenic CCR6^+^ Th1 subsets such as found in MS patients [12]. Importantly, CD45+ cells were found to accumulate within the conjunctive stroma of the choroid plexus in CCR6-deficient mice after MOG immunization, hinting at a role for this particular chemokine-chemokine receptor pair in the transepithelial migration step in EAE [11].

T-cell trafficking via the choroid plexus may be amplified in various neuroinflammatory and neuroinfectious diseases characterized by elevated CSF levels of chemokines (e.g. [13,14]). Spatiotemporal analyses of the pathogenesis of murine and rodent experimental autoimmune encephalomyelitis indicated that periventricular structures are among the primary target areas of early T-cell infiltration [10,15]. Migration of T-cells into the CSF via the choroid plexus may similarly contribute to the preferential localization of focal demyelinated plaques in periventricular areas in patients with multiple sclerosis [16,17].

**Fig 1:**
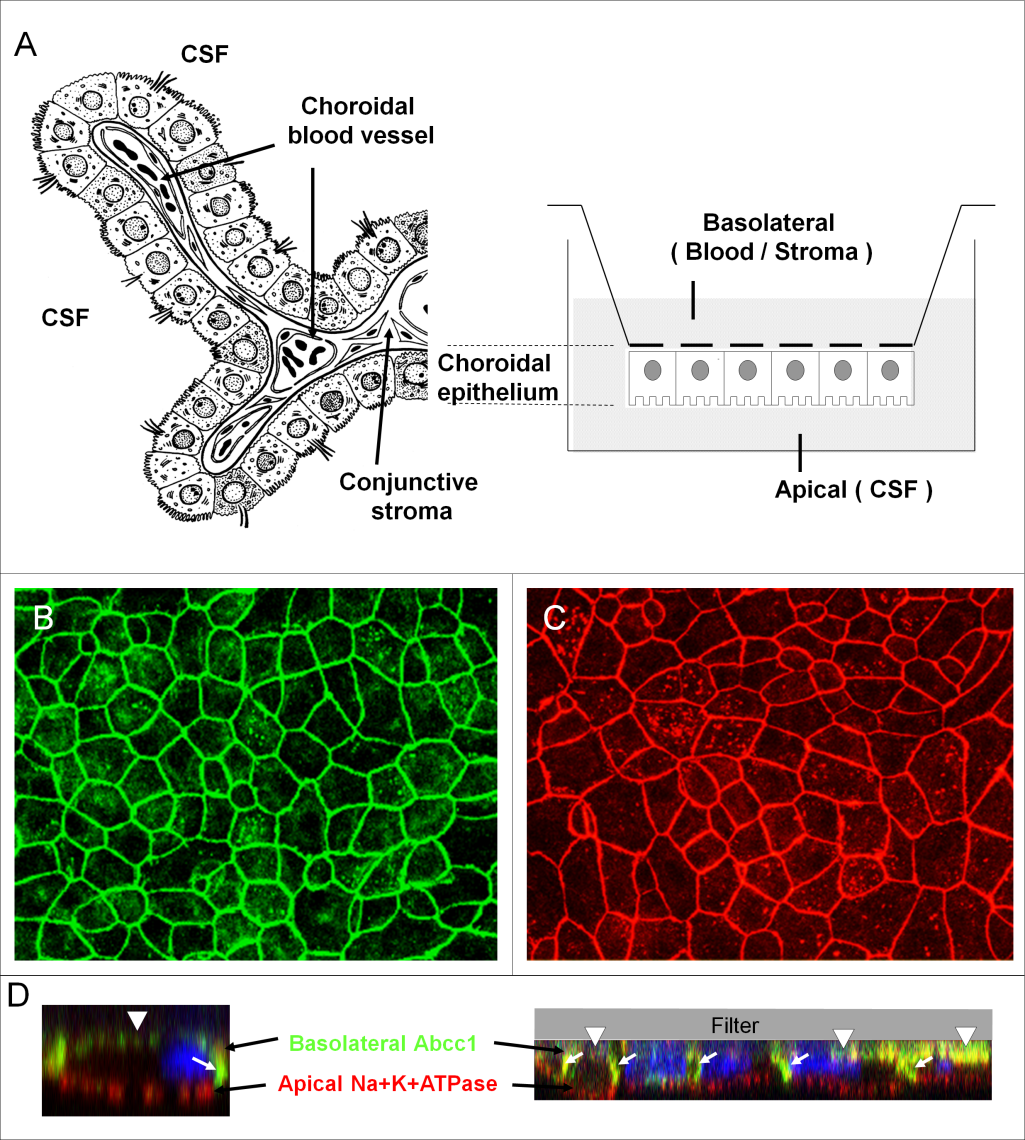
The cellular model of the blood-CSF barrier. (A) Schematic representation of a choroidal villus and of the experimental set up illustrating the two-chamber culture device. Left: the choroidal epithelium which forms the actual tight barrier controlling access into the CSF [18] delimits a stroma, in which the fenestrated vessels lacking a typical blood-brain barrier phenotype, express P-selectin [4]. Right: The epithelial cell monolayer grown on the lower side of the filter separates the upper chamber corresponding to the blood/stromal or basolateral space, from the bottom chamber representing the CSF or apical compartment (adapted from [18,41]. (B and C) Immunofluorescent staining of occludin (B) and claudin 1/3 (C) showing a typical intercellular distribution of the tight junction proteins in the confluent inverted monolayers of choroidal epithelial cells. (D) Immunofluorescent staining of Na^+^K^+^ ATPase and ABCC1, showing the expected respective apical and basolateral membrane localization in the choroidal epithelial cells. The left image is a close up of a single cell to better appreciate the polarity of distribution of the 2 proteins. Nuclei appear in blue. Arrows show the lateral cellular membranes best seen in the z direction by confocal analysis, arrowheads show the basal labeling of ABCC1.

As in many epithelial barrier sites, cell recruitment across the choroid plexus is a two-step process. It first involves endothelial extravasation across the choroidal vessels leading to cell accumulation in the choroidal stroma and then implies cell trafficking from the stroma to the CSF space across the choroidal epithelium (Fig 1A), which constitutes the actual anatomic site of the blood-CSF barrier [18]. Until recently, the lack of an adequate technology impeded investigation of the second step. In this paper, we established a differentiated cellular model of the rat blood-CSF barrier dedicated to the study of cell migration. We investigated the migration of activated T cells across the choroidal epithelium, its impact on the integrity of the tight junctions, and its potentiation by chemokines involved in the physiopathology of neuroinflammatory diseases.

## Material and Methods

### Animals

Animal care and procedures were conducted according to the guidelines approved by the French Ethical Committee (decree 87–848) and the European Community directive 86-609-EEC. OFA timed pregnant female and adult male rats were obtained from Harlan (Gannat, France). The animals were kept in an individually ventilated caging system with appropriate bedding for pregnant females, in a temperature controlled room with a light-dark cycle of 12/12 hrs, and with food and water ad libitum.

### Choroidal epithelial cell monolayers

Primary cultures of epithelial cells from choroid plexuses sampled on two day-old rats were prepared using a modification of the method described by Tsutsumi and coll. [19]. Following rat pup sacrifice by decapitation, brain was removed from the skull. Choroid plexuses from both lateral ventricles were rapidly dissected under a stereo-microscope and kept in prewarmed (37°C) culture medium consisting of Ham’s F-12 and DMEM (1:1) supplemented with 10% (v/v) fetal calf serum, 2 mM glutamine and 50 μg/ml gentamycin (all reagents from Gibco, Life Technologies SAS, Saint-Aubin, France). Plexuses were rinsed twice in PBS and incubated in PBS containing 1 mg/ml pronase (Sigma, L’Isle d’Abeau Chesnes, France) for 25 min at 37°C. Predigested plexuses were recovered by sedimentation and washed once with PBS. The supernatant containing mostly single non-epithelial cells was discarded and the large clumps of epithelial cells were briefly incubated in 0.025% trypsin (Gibco) containing 12.5 μg/ml DNase I (Roche Diagnostics GmbH, Mannheim, Germany). The supernatant resulting from sedimentation was withdrawn and kept on ice, with 10% fetal calf serum. Fresh trypsin solution was added to the tissue and this step was repeated five times. Cells were pelleted by centrifugation at 800 g for 5 minutes and pellets were resuspended in culture media. Epithelial cells were further enriched by differential attachment on plastic dishes. After 2 hours of incubation at 37°C resulting in fibroblast, endothelial cell, and macrophage adhesion to the plastic, the cell suspension was collected. Cells were seeded on Transwell-Clear filter inserts (6.5 mm diameter, 0.33 cm^2^ surface, 3.0 μm pore size, Corning B.V. Life Sciences, Amsterdam, The Netherlands) at a density of 0.65 cm^2^/choroid plexus as follows. Inserts were precoated on their lower side with laminin (Becton Dickinson, Le Pont-de-Claix, France) as described by the manufacturer. Cells were allowed to attach on inverted filters covered with culture medium at 37°C in a 5% CO_2_ atmosphere before inserts were positioned in a normal orientation in multiwell plates. Culture medium was added in both chambers, changed every other day, and renewed 24 hr prior to the migration studies. It was supplemented with 5 μg/ml insulin, 5 μg/ml transferrin, 5 ng/ml sodium selenite, 10 ng/ml epidermal growth factor, 2 μg/ml hydrocortisone, 5 ng/ml basic fibroblast growth factor and 500 μM hypoxanthine. Experiments were performed 5 days after confluence. Cell-free laminin-coated inserts were kept in the same conditions.

### Preparation, characterization, and labelling of T cells

#### Splenocyte isolation

The spleen was removed aseptically from Sprague-Dawley rats and placed in RPMI medium 1640 supplemented with 10% FCS, 50 μM 2-mercaptoethanol, 1% sodium pyruvate, 1% non-essential amino acids, 1% L-glutamine, 1% penicillin, 1% streptomycin, (all reagents from Gibco). The spleen was cut in small fragments which were forced through a cell strainer (mesh size of 200 μm, Netwell insert, Corning). Cells were pelleted by centrifugation and suspended in 1 ml of erythrocyte lysis buffer (0.15 M NH_4_Cl, 10 mM NaHCO_3_, 0.1 mM Na_2_ EDTA). Cells were kept at room temperature (20–24°C) for 3 minutes with occasional gentle shaking and the lysis buffer was neutralized with 10 ml of culture medium. Cells were pelleted, washed once with culture medium, and resuspended in the same medium at a density of 5 x 10^5^ cells per well. T cell proliferation was induced by phytohemagglutinin (PHA, 1 μg/ml, Sigma Aldrich, St-Louis, MO) or concanavalin A (ConA 1.2 μg/ml Sigma Aldrich) for 72 hours. Activated T cells were further cultured at a density of 2 x 10^5^ cells/ml for 6 days in the presence of recombinant IL-2 (20 UI/ml; Euromedex, France).

#### T cell characterization

T cells were pelleted by centrifugation and resuspended in staining buffer (PBS supplemented with 2% FCS and 0.1% (w/v) sodium azide). Following the addition of mouse anti-rat CD3 (Clone G4.18, BD Pharmingen, Le Pont de Claix, France), cells were incubated on ice for 20 minutes. They were washed with 2 ml of staining buffer and incubated with FITC-conjugated goat anti-mouse IgG (Star70, Serotec France, Cergy Saint-Christophe, France) for 20 minutes on ice. Following washing in staining buffer, cells were resuspended in 10 mM EDTA containing PBS and kept on ice until analyzed by flow cytometry (Coulter Epics XL, Beckman Coulter, Villepinte, France). Staining with the mouse isotype IgG_3_, κ (BD Pharmingen) was performed as a negative control. Cell viability was assessed by propidium iodide exclusion. Propidium iodide was added to a final concentration of 10 μg/ml, prior to flow cytometry analysis. Viability was superior to 98%, and 95% of the cells were CD3 positive.

Chemokine receptor expression was evaluated by mRNA analysis in activated T cells in comparison to rat splenocytes. Total mRNA was extracted and DNAse-treated using RNeasy^®^ Mini Kit (Qiagen, Courtaboeuf, France), according to the manufacturer’s instructions. RNA was reverse-transcribed using random hexamer DNA primers and Moloney Murine Leukemia Virus reverse transcriptase (Invitrogen, Life Technologies SAS), according to the manufacturer's instructions. Selected chemokine receptor cDNAs were amplified using Hot start Taq polymerase (Qiagen) and the primers listed in Table 1. β-actin was amplified using 5’-GGGACCTGACAGACTACCTCATG-3’ and 5’-GAGGATGCGGCAGTGGC-3’ as forward and reverse primers, respectively. Cycle parameters were as follows: 45 s at 94°C, 60 s at 59 or 60°C and 90 s at 72°C. The number of PCR cycles was adjusted for each gene in order to yield conditions of near-linearity between the amount of cDNA added in the PCR reaction and the amplified DNA band intensity estimated on agarose gels using the Gene Tools Analysis Software version *3*.*02*.*00* (SynGene, Cambridge). Chemokine receptor signals were normalized to β-actin signals and used to generate semi-quantitative data of chemokine receptor mRNA.

**Table 1:** Chemokine receptor expression in activated T cells. Total mRNA was prepared from T cells obtained after PHA- or ConA stimulation and cultured in the presence of IL-2. Semi-quantitative RT-PCR was run using the listed specific primers, and scoring of–to +++ was attributed based on the level of chemokine receptor mRNA in cells versus control rat spleen, all levels being normalized to that of beta-actin. ++ indicates levels comparable to that observed in the spleen.

Chemokine receptor	Chemokine ligand	Primer sequences	Level of expression in stimulated lymphocytes (versus rat spleen)	
			PHA	ConA
CCR2	CCL2	5’-CCACCACACCGTATGACTATGAT-3’	++	++
	(MCP-1)	5’-ACAGCATGGACAATAGCCAAATA-3’		
CCR5	CCL5	5’-TGTTTCGCTGTAGGAATGAGAAG-3’	+++	++
	(Rantes)	5’-GTTTGACAATGTGTTTTCGGAAG-3’		
CCR7	CCL19 &	5’-TGGTCATTTAGGTGTGCTT-3’	++	++
	CCL21	5’-AGCAGGTAGGTATCCGTCATGG-3’		
CXCR3	CXCL10	5’-TTCCTCTGTTCACGGCAAGT-3’	+	+/-
	(IP-10)	5’-TGGAGCAGGAAGGTGTCTGT-3’		

#### T cell labeling

On the day of the migration experiment, T cells were labeled with the cell permeant 5-(and -6)-carboxyfluorescein diacetate, succinimidyl ester (CFSE, Molecular Probes, Life Technologies), used for its strong and steady labeling property, and minimal toxicity [20]. Cells were thoroughly resuspended in culture medium. CFSE (5 mM stock solution in DMSO) was added to a final concentration of 15 μM. Cells were protected from light and incubated for 30 minutes at 37°C. Cells were washed twice in a large volume of culture medium and resuspended in culture medium at a concentration of 2 x 10^6^ cells/ml.

### Transwell migration assay

**Fig 2:**
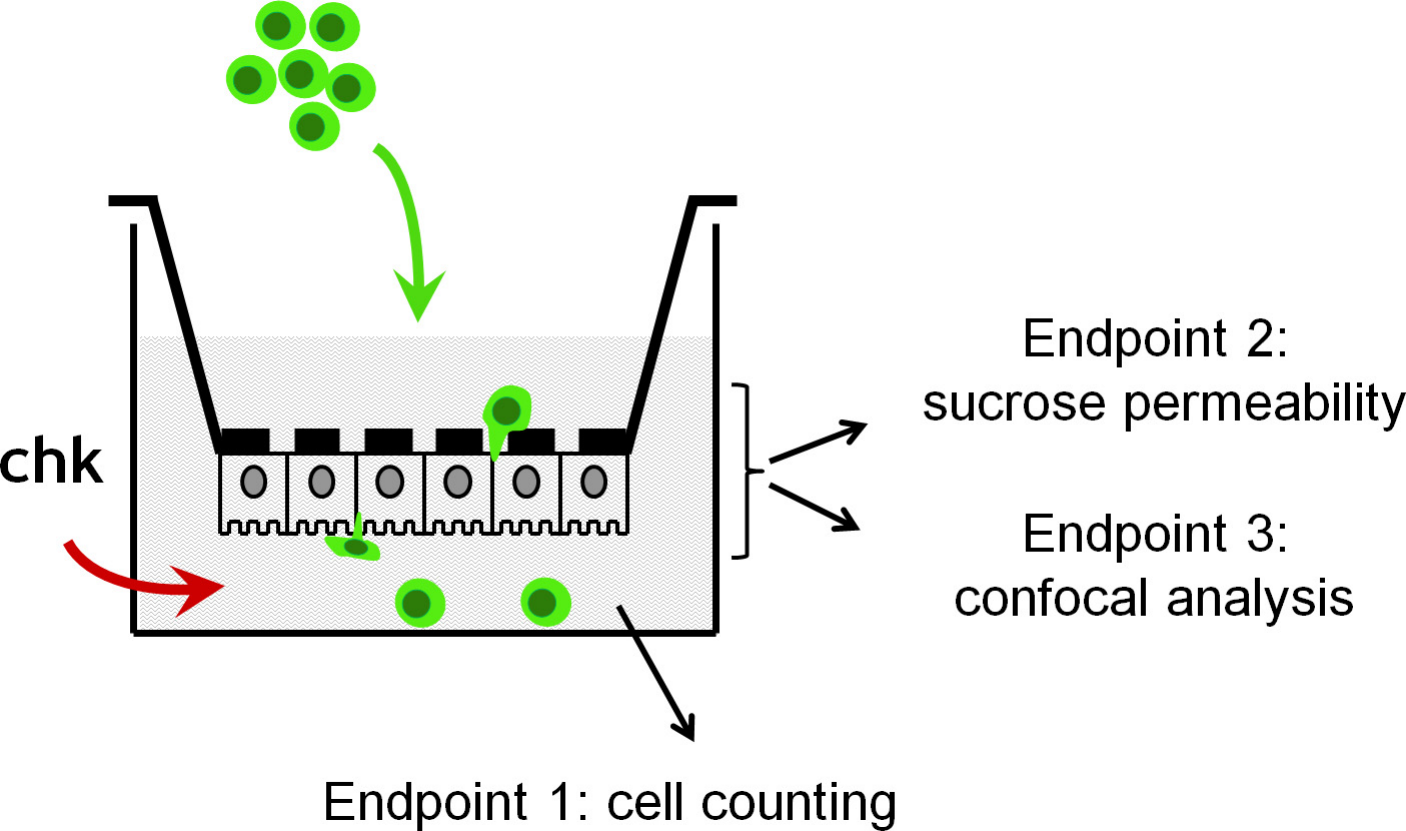
Schematic representation of the Transwell migration assay. Fluorescently labeled lymphocytes (10^6^ cells/ml) are added into the upper chamber, in the presence or absence of chemokines in the lower chamber. Counting of cells retrieved from the lower chamber enables to determine the percentage of lymphocytes that fully migated through the choroidal epithelial cells. Staining protocols of the cell covered filters following the migration study allow defining the localization of lymphocytes within the filter/epithelium system, and approaching the route of transmigration.

Labeled lymphocytes were diluted two-fold in the upper chamber of epithelial cell-covered Transwell inserts or cell-free inserts, to yield a final concentration of 10^6^ cells/ml (250 10^3^ cells/filter). The recombinant chemokines CCL5 (100 ng/ml), CCL2 (20 ng/ml), and CXCL10 (20 ng/ml), all from R&D Systems (Lille, France) were added in the lower chamber of some filters. After 7 hours of migration at 37°C in 5% CO_2_, T cells were collected by centrifugation of the lower chamber medium and numbered by fluorescent microscopy using a cell counting chamber (Fig 2).

### Immunocytochemistry

Immunocytochemical staining of tight junction proteins occludin, claudins 1/3, claudin 2, Na^+^K^+^ATPase and ABCC1 in choroidal monolayers was performed as follows. The cell-covered filters were briefly washed with ice-cold Dulbecco’s PBS containing Ca^2+^ and Mg^2+^ (Invitrogen), and monolayers of choroidal epithelial cells were fixed by methanol/acetone (1/1) for 90 s. After fixation, filters were washed 3 times on ice with Dulbecco’s PBS without Ca^2+^ and Mg^2+^, and stored at 4°C until processed for immunocytochemical staining. The distribution of tight junction proteins was studied using rabbit polyclonal antibodies raised against occludin (#71–1500, 0.625 μg/ml), claudin 2 (#51–6100, 1.25 μg/ml), and claudins 1/3 (#71–7800, 1.25 μg/ml, all antibodies from Life Technologies). The localization of Na^+^K^+^ATPase and ABCC1 was studied using a mouse monoclonal antibody (NB300-146, Novus Biologicals, diluted 1/1000) and a rabbit polyclonal antibody (ALX-210-841-C100, Alexis Biochemicals, 2,5 μg/ml), respectively. The choroidal monolayers were saturated in 10% FCS containing PBS for 1 hour at room temperature. The primary antibodies were diluted in the same buffer and incubated with choroidal monolayers overnight at 4°C. After 3 washes in PBS, the filters were incubated for 2 hours at room temperature with Alexa Fluor 488 (#A-11008) or Alexa Fluor 546 (#A11-010) conjugated anti-rabbit IgG goat antibodies (Molecular Probes, Life Technologies) used at a dilution of 1/1000 in FCS containing PBS. After washes in PBS, cell-covered filters were mounted on slides with Fluoprep (Biomerieux, Lyon, France) and examined using a Leitz DMRB fluorescence microscope or a Leica TSC-SP confocal scanning head mounted on an upright Leica DMR microscope (Leica Microsystèmes SAS, Nanterre, France). Staining without primary antibody was run as negative control.

### Permeability studies

The transport of phenol red was measured as an index of active organic anion efflux (from CSF to blood) across the choroid plexus to assess choroidal differentiation. It was also used as a visual criterion to readily detect defective monolayers that would not display an imbalance in phenol red concentration (i.e. red color intensity) between the apical and basolateral compartments. Precise volumes of culture medium containing 23 mM of phenol red, (i.e. the concentration in commercially available DMEM/F12 medium), were added to the upper and lower compartments of the inserts. Both media were collected between 17 and 47 hours after addition. One hundred μl of medium was diluted with 300 μl of 0.5N NaOH and the spectrum of the solution was recorded between 500 and 700 nm in a Carry 100 dual spectrophotometer (Varian-Agilent Technologies, Massy, France). The difference in optical densities between 550 and 700 nm is proportional to the concentration of phenol red in the medium. The active clearance of phenol red was calculated as the volume cleared from the basolateral to the apical chamber during the incubation period, using the following equation:

$$Cl=[C_{a}-C_{b})V_{a}V_{b}]/[C_{a}V_{a}+C_{b}V_{b}]$$

where C_a_ and C_b_ are the concentrations at the time of sampling in the apical (lower) and basolateral (upper) solutions, respectively, and V_a_ and V_b_ are the volumes of the apical and basolateral solutions, respectively. Final data expressed in μl·cm^-2.^hr^-1^ were obtained by dividing the clearance value by the surface area of the monolayer and the duration of the transport [21].

The monolayer permeability to [^14^C]-sucrose (350 mCi/mmol, Amersham, Little Chalfont, England) was measured at the end of the transmigration assay to evaluate the effects of T-cell migration on the paracellular gate function of the choroidal cells. Culture inserts were rinsed once on both sides with Ringer-Hepes buffer (RH, 150 mM NaCl, 5.2 mM KCI, 2.2 mM CaCI_2_, 0.2 mM MgCl_2_, 6 mM NaHCO_3_, 2.8 mM glucose, 5 mM Hepes, pH 7.4) prior to the permeability study. Fresh RH was added to both compartments of the insert and volumes were adjusted to reach level equilibrium in order to prevent any hydrostatic pressure. Sucrose (0.18 nCi/μl in RH) was added to the upper chamber, and at regular intervals thereafter, the insert was transferred to another well in order to minimize the backflux of molecules from the lower to the upper chamber. Laminin-coated filters without cells were run similarly. All incubations were performed on a rotating platform (200 rpm) at 37C. Radioactivity was determined by liquid scintillation counting using a TRI-CARB 1600-TR analyzer (Packard, Canberra, NZ) in aliquots sampled from the upper chamber and the successive lower wells. Permeability coefficients were calculated as described in details [21,22]. Briefly, the flux of material across the monolayer was estimated as the amount cleared from the donor fluid [23]. The volume clearance is given by the following equation:

$$\mathrm{Volume cleared}=C_{a}V_{a}/C_{d}$$

where C_a_ is the concentration in the acceptor lower solution at the time of sampling, V_a_ is the volume of the acceptor solution, and C_d_ is the concentration in the donor upper solution. The latter was corrected for each sampling period by adjusting its value for the amount of molecule cleared during the previous time point. During the course of the experiment, the clearance volume increased linearly with time. The rate of clearance, equal to the slope of a plot of the cumulative volume over time, was determined by least square regression analysis. As C_d_, is assumed constant over each sampling period, and the backflux is considered negligible, the rate of clearance becomes equal to the permeability-surface area product (PS in μl.min^-1^.filter^-1^). The reciprocals of the PS products of the serially arranged layers composing the cell monolayer-laminin-filter system are additive [23] and verify the following equation:

1/PSt=1/PSf+1/PSe

where PSt and PSf are the PS products determined for filters with and without epithelial cells, respectively, and PSe is the permeability-surface area product of the epithelial monolayer. The permeability coefficient of the epithelial cells Pe (cm.min^-1^) was obtained by dividing PSe by the surface area of the filter.

Electric resistance was measured across cell-covered filters using an EVOM resistance meter (WPI, Hitchin, UK). The value obtained was corrected by subtracting the value measured across laminin coated filters without cells. The resulting resistance value was multiplied by the surface area covered by the cells on the inverted filters (0.4 cm^2^), to generate transepithelial electric resistance (TEER) expressed as ohm.cm^2^.

## Results

**Fig 3:**
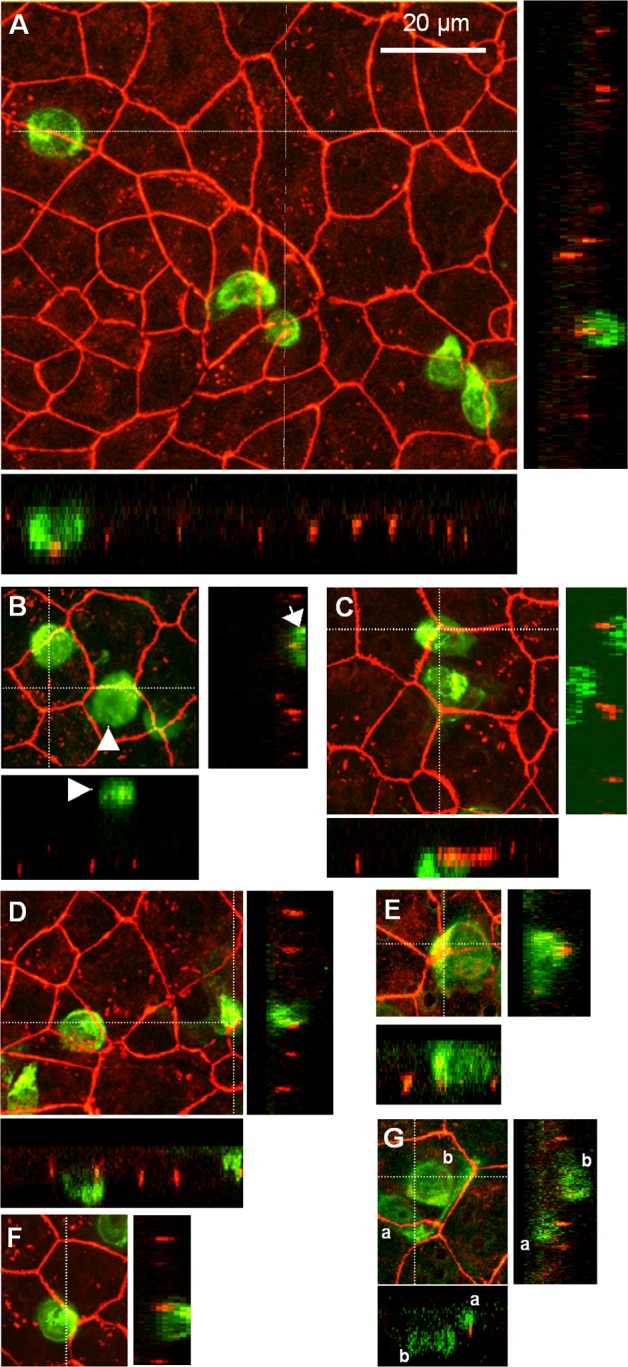
Distribution of claudins and activated T cell localization following transmigration assay across the choroidal epithelium. T cell adhesion to and migration across the epithelium do not cause any alteration in the continuous peripheral distribution of epithelial tight junction proteins claudin 1/3 and 2. Stacks of optical Z-sections obtained by confocal microscopy across the whole filter + epithelial layer allowed visualizing all T cells associated with the system. Their relative position with respect to the tight junctions revealed by claudin1/3 (A, B, D, G) or claudin 2 (C, E, F) immunocytochemistry was determined in xz or yz views and used to discriminate transmigrated immune cells adhering to the apical membrane from those still located basolaterally or remaining in the upper chamber (example in B). Activated T cells contacting either the basolateral or the apical membrane domain of the epithelium are often located at tricellular corners (A, C, E, F) hinting at a paracellular route of T cell migration across the monolayer. Most T cells present a non-homogeneous fluorescence intensity or a typical comma-like shape with a uropod and a cellular protrusion that could be a leading edge (stars in A, D), which reflect their motility. In G, the T cell “a” extends a long cytoplasmic projection seemingly scanning the intercellular junction towards the tricellular corner.

The choroidal epithelium reconstituted in an inverse configuration reproduced barrier features typical of the blood-CSF interface in vivo, similar to those reported in details for the normal configuration [21,22]. It formed a hydrodynamic barrier maintaining imbalanced fluid levels between the two chambers (not shown). Staining of the tight junction transmembrane proteins occludin, claudin 1/3 (Fig 1B and 1C), and claudin 2 (Fig 3C, 3E and 3F) demonstrated a continuous immunoreactivity along epithelial cell borders. The cell monolayer displayed a highly restricted paracellular diffusion indicated by an epithelial permeability to sucrose of 0.314 ± 0.050 x 10^−3^ cm.min^-1^ (n = 11 from 4 cell preparations). The TEER was of 134.0 ± 2.7 ohm.cm^2^, and was similar to the moderate TEER measured in vivo [24,25]. This value is consistent with the expression and membrane localization of the pore-like forming claudin-2 (Fig 3C), a tight junction protein responsible for low electrical resistance in epithelia. Finally, the cells reproduced the specific property of the blood-CSF barrier in vivo to actively transport phenol red out of the brain [26], as demonstrated by the monolayer capacity to transport this organic anion from the apical to basolateral compartment against the building concentration gradient (active clearance of 13.9 ± 2.7 μl.cm^-2^.hr^-1^, n = 13 from 3 cell preparations). This functional evidence of epithelial polarization was further substantiated by the respective apical and basolateral localization of Na^+^K^+^ ATPase and ABCC1, two proteins involved in energy-dependent transport functions at the blood-CSF barrier (Fig 1D).

**Fig 4:**
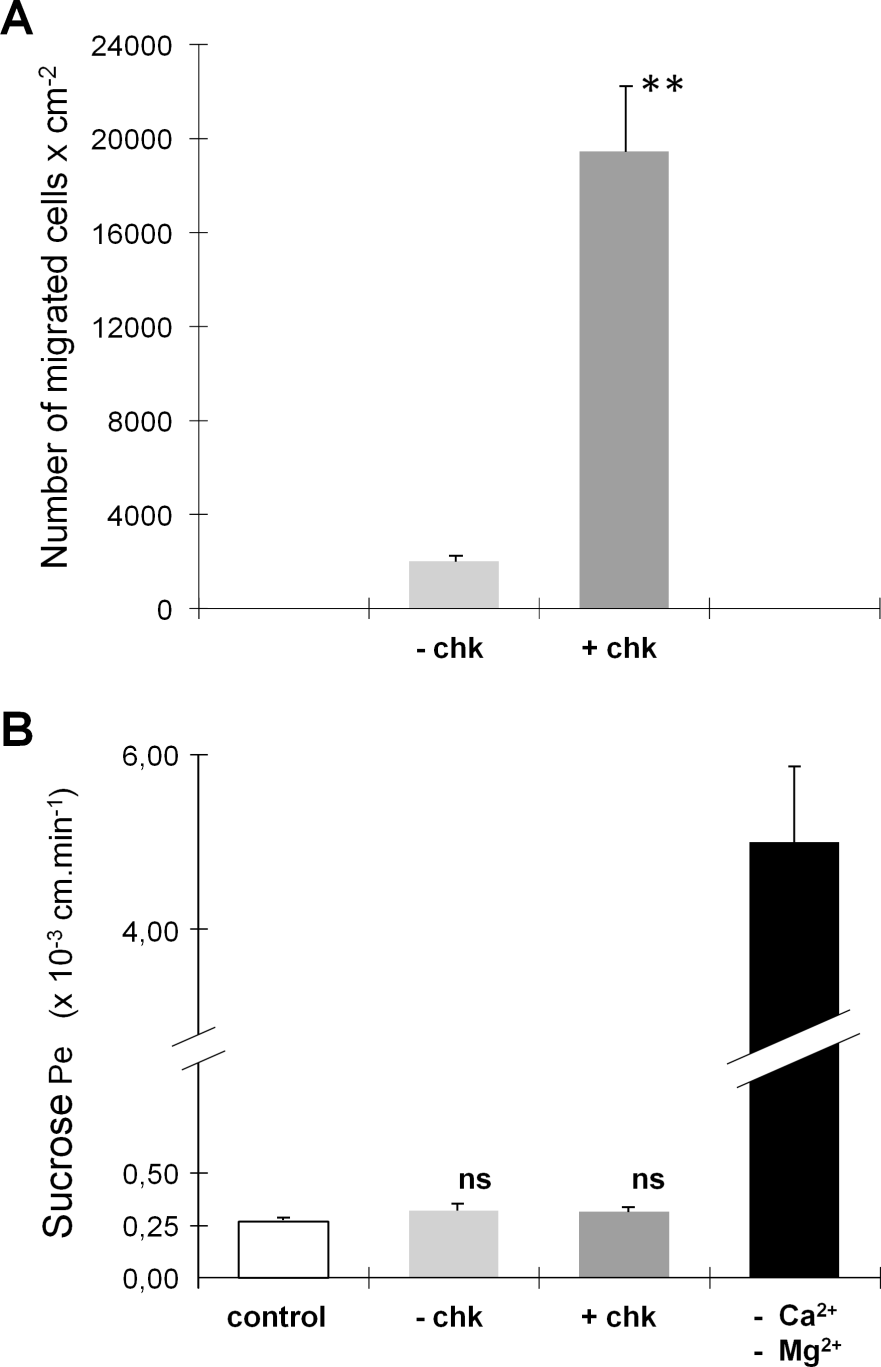
Activated T cell migration across the choroidal epithelium. (A) Transepithelial chemotaxis of PHA-activated T cells in response to CCL2, CCL5 and CXCL10. **: statistically different from migration without chemokines p<0.01, two-tail Student's t test for unequal variance. The migration indices, measured after a 7-hour period, represent means ± SD, n = 3 or 4. (B) T cell chemotaxis does not alter the paracellular gate function of the blood-CSF barrier. Epithelial permeability coefficients for the paracellular marker sucrose are calculated and expressed in cm.min^-1^ [21]. ns: not statistically different from control group (without T cells) p<0.05, one-way ANOVA followed by ‘a posteriori’ Dunnett’s test. Data represent means ± SD, n = 3.

**Fig 5:**
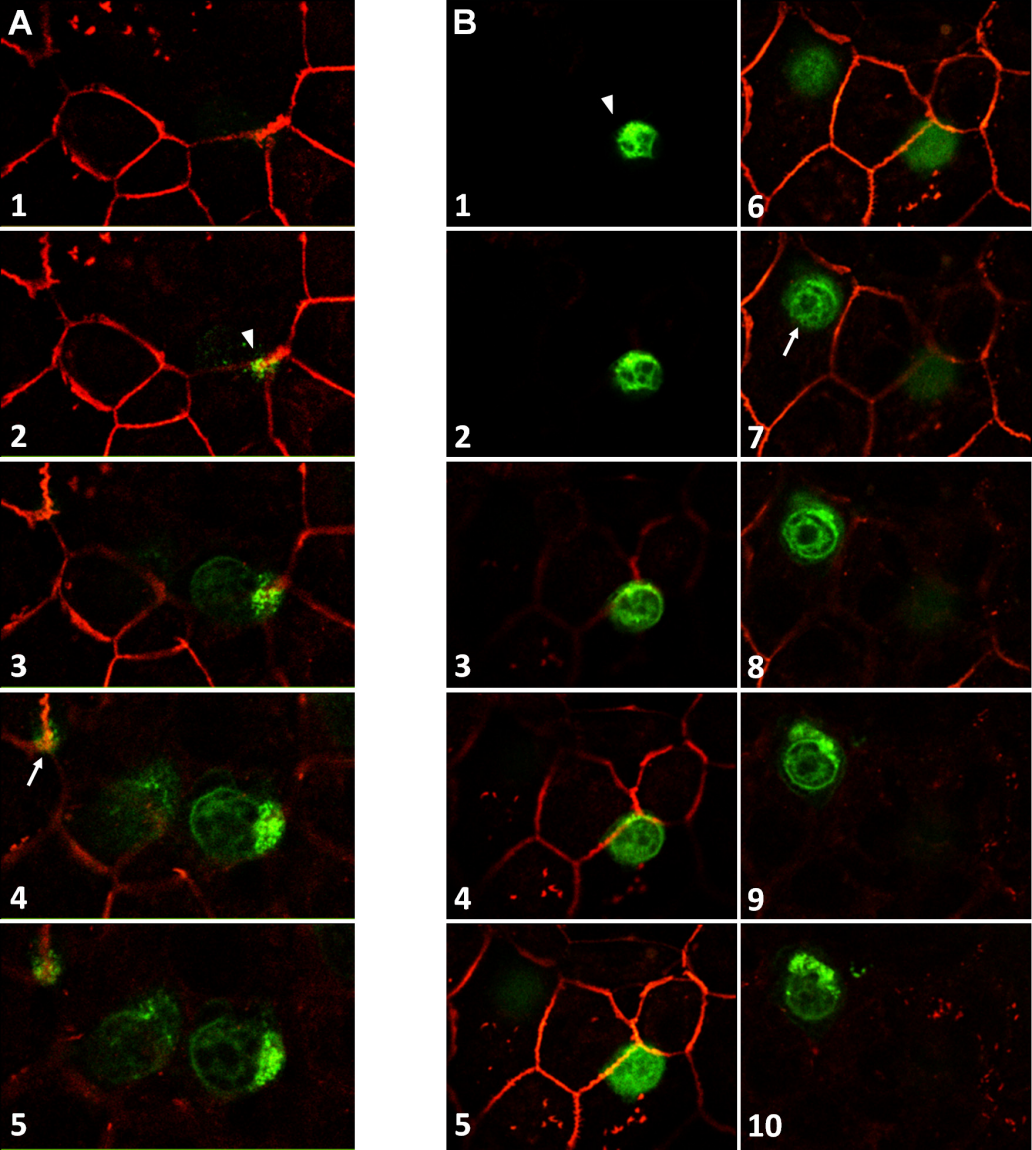
Confocal analysis of T cell transmigration route. Successive optical Z-sections through the filter + epithelial cell system are represented in an apical to basal order. (A) The most apical confocal plane is at the level of the tight junction protein claudin 2. The T cell, barely visible in this section, emerges on the following plane at a tricellular corner (white arrowhead). The leading edge is then visualized in section 3, followed by the cell body in more basal focal planes. A second, presumably transmigrating T cell similarly appears at another tricellular corner (Section 4, white arrow). (B) The T cell visible in the first confocal plane (white arrowhead) has migrated through the epithelial monolayer. It is located apically relative to the sections displaying the tight junction protein claudin 2 (visible from section 3), and associated with a tricellular corner (Section 5), through which it may have migrated. A second T cell (Section 7, white arrow) is visible in the second half of the plane series. It is mostly present in sections that are basal to the tight junction network.

The activated T cells prepared for transmigration experiments expressed gene transcripts for the chemokine receptors CCR2, CCR5, CCR7, and CXCR3 to a lesser extent (Table 1). Accordingly, these cells were responsive to the attracting effect of the corresponding ligands CCL2, CCL5, and CXCL10, as assessed in a chemotaxis assay across cell-free laminin-coated filters. The number of migrated cells per cm^2^, measured over a 30-minute period, were 4470 ± 435 and 29390 ± 8250, in the absence and presence of chemokines, respectively. Migration of T cells across choroidal epithelial cell monolayers in the absence of exogenous chemokines was restricted but not fully abrogated by the cellular barrier, representing for example 2.5% of the migration observed across cell-free laminin-covered filters in the case of PHA-treated cells. Addition of the chemokines CCL2, CCL5, and CXCL10 to the lower apical chamber stimulated T cell migration. It resulted in a 10-fold increase in the migration index of PHA-treated cells (Fig 4A). A similar effect of chemokines was observed on the migration index of ConA-treated cells (7-fold increase, data not shown). Transmigrated cells were controlled to be CD3 positive by facs analysis. Transmigration and chemotaxis of activated T cells did not affect the functional and structural integrity of the choroidal epithelial barrier. The flux of sucrose across the choroidal epithelium was not altered following transcellular migration of activated cells, in contrast to the 19-fold increase observed when tight junction disassembly was induced by Ca^2+^/Mg^2+^ removal (Fig 4B). Immunofluorescent staining of claudins performed after the chemotaxis period did not reveal any visible alteration in the continuous distribution of these proteins at cell borders (Fig 3A–3G). Confocal analysis enabled to determine the position of T cells relative to claudin-containing tight junction complexes (Figs 3 and 5). Resisting the brief washing step before fixation, a few cells possibly engaged into migration through the pores were found still adhering to the filter membrane in the upper chamber (Fig 3B, arrowhead). Many cells were located between the filter and the epithelial cell monolayer, appearing in focal planes that were basal to the tight junction complexes (e.g. Figs 3C, 3E, 3G, 5A and 5B). Some of these cells which displayed a cellular extension in contact with the junctional complexes had apparently initiated transepithelial migration (Fig 5A). Finally, a few other cells were observed associated with the apical surface of the epithelial cells (e.g. Fig 5B). Regardless of their position (apical or basal relative to tight junction complexes), 70% of migrating T cells appeared to contact interepithelial cell borders and 30% were localized at tricellular corners (e.g. Fig 5A and 5B and Table 2). In Fig 3G, one T cell, positioned basally relative to claudin immunoreactivity, extends a long cytoplasmic process seemingly scanning the intercellular junction towards a tricellular corner. This particular pattern of T cell association with the junctions supports a paracellular route of migration across the blood-CSF barrier and suggests that intersections between three or more neighboring cells represent preferential sites of cell infiltration.

**Table 2:** Quantification of migrating T cell in contact with interepithelial tight junctions. T cells that had reached the epithelial monolayer and were in contact with the interepithelial tight junctions (identified by claudin1/3 and claudin2 immunostaining) were quantified on confocal images through observation of all xy, xz and yz plans. Results are expressed as the percentage of total number of cells analyzed. Data are mean ± SEM, n = 12 monolayers from two 2 choroidal cell preparations.

	No TJ contact	TJ contact	
% of cells	27.5 ± 4.7	72.4 ± 4.7	
		Bicellular TJ contact	Tricellular TJ contact
		38.1 ± 5.9	34.4 ± 4.7

## Discussion

Trafficking of immune cells across epithelial barriers, from the stromal or mucosal space to the luminal space, occurs in a number of organs such as lung, kidney, or intestine. Addressing transepithelial migration mechanisms in vitro requires the establishment of cellular barriers in an inverse setting that enables to expose epithelial cells to immune cells via their basolateral membrane domain. Such inverse models of the bronchial epithelium [27] or the intestinal epithelium [28] have been successfully developed and used to investigate cell trafficking in these peripheral organs. Analogous models of the porcine and murine choroidal epithelium were recently introduced to explore the involvement of the blood-CSF barrier in *Streptococcus suis* meningitis and subsequent polynuclear neutrophil recruitment, [29,30], and in monocyte recruitment following brain injury [31]. T Lymphocyte migration has also been documented across a model derived from human malignant choroid plexus papilloma cells [32]. To circumvent the lack of methodology allowing the visualization of lymphocyte migration across the blood-CSF barrier in vivo in rodent, we have now established an inverse cellular model, based on primary culture of rat choroid plexus epithelial cells, which displays structural and functional features of the choroidal barrier in vivo. It reproduces the continuous circumferential localization of various tight junction transmembrane proteins known to confer paracellular barrier properties. The functionality of these sealing proteins is demonstrated by a low permeability of the monolayer to the small polar compound sucrose, by a TEER similar to those measured in vivo, and by the cell ability to maintain an imbalance in phenol red concentrations between the apical and basal chambers. The capacity of the cell monolayer to create this imbalance by mediating the unidirectional transport of phenol red substantiates one hallmark of epithelial barriers, which is cell polarization. This property of the *in vitro* blood-CSF barrier, previously described in vivo for rabbit choroid plexuses [26], also indicates that a differentiated choroidal phenotype is maintained in the cells cultured in an inverse configuration. This was further substantiated by the polarized localization of two landmark choroidal proteins, Na^+^K^+^ ATPase and ABCC1, respectively at the apical and basolateral membranes of the cells. These monolayers constitute an appropriate tool to investigate the process of immune cell trafficking across the blood-CSF barrier.

We have used this differentiated in vitro model to explore the migratory behavior of activated T cells across the resting choroidal epithelium. T cell recruitment through the choroid plexus implies a multistep process, involving the initial extravasation of immune cells through the choroidal endothelium, followed by their transepithelial migration from the stroma into the ventricular space (Fig 1A for a description of the anatomical organization of a choroidal villus). These two steps fundamentally differ in that shear forces imposed on immune cells by the blood flow in the former do not apply in the latter step. Therefore, transepithelial migration studies do not require the implementation of dynamic flow-based transmigration assays, and should be performed in static conditions. We observed a spontaneous migration of T cells across the cellular barrier that largely increased following addition into the CSF compartment of exogenous chemokines for which the cells expressed the corresponding receptors (Fig 4). Chemokine synthesis and secretion by the choroid plexus has not yet been investigated extensively. Current knowledge indicate that naïve choroid plexuses constitutively produce and secrete some chemokines, as shown by either gene expression studies, or at the protein level by immunochemistry, ELISA, or protein array analysis ([4,11,33,34,35], and unpublished results). Choroidal epithelial cells in culture retain this capacity, and in the absence of stimulation, produce and secrete chemoattractant agents in a polarized way [34,35]. In our experimental conditions whereby the resting epithelial cells were allowed to secrete basal levels of endogenous chemokines prior to T cell addition, chemotaxis may have contributed to the apparently spontaneous migration process. Activated T cell trafficking was substantially increased upon addition of a mix of CCL2, CCL5, and CXCL10, three chemokines whose receptors are expressed by these immune cells. Our results indicate that recruitment of stromal activated lymphocytes into CSF may be stimulated in various neurological infectious or inflammatory diseases, for which increased CSF levels of these chemokines have been reported [13,14].

The data we generated using PHA and CON-A activated T cells, have heuristic value to understand the blood to CSF trafficking of other T cell subsets displaying specific determinants. For instance, T cells in human CSF are mostly memory cells and display a restricted repertoire of chemokine receptors, which is similar between patients with non-inflammatory and inflammatory neurological disorders [3,36]. The co-expression of CCR7 with CXCR3 and CCR5 in these cells indicates that they can be recruited by both the homeostatic lymphoid chemokines CCL19 or CCL21, and by inducible chemokines such as CXCL10 or CCL5. Evidence for CCL21 synthesis in epithelial cells of human choroid plexuses from both control individuals without CNS inflammation and multiple sclerosis patients [33], could therefore be of relevance to the migration of T cells across the blood-CSF barrier in neuroimmune surveillance. The choroidal epithelium produces another constitutive chemokine, CCL20, the only ligand known for CCR6 [11,37]. T cells that recognize processed MOG are present almost exclusively among CCR6+ memory ⁄ effector T cell subset in human [12]. Mice lacking this receptor CCR6 are highly resistant to the induction of EAE, due to the inability of CCR6- T_H_17 pathogenic cells to enter the inflamed brain and initiate the disease. The high expression of CCL20 in choroidal cells and the concurrent accumulation of CCR6- cells in the choroidal stroma of deficient mice suggest that the early migration of pathogenic cells occurs through this interface and is dependent on the interaction between CCL20 and CCR6.

CNS-specific trafficking determinants have not been identified yet. Selectivity in chemokine transport and presentation may be involved in tissue-specific variations in leukocyte homing mechanisms, as reported recently for CXCL10 and CCL2 in human vascular endothelial cells of various tissues [38]. Insights into CSF-homing mechanisms will be provided by precisely defining the repertoire of chemokines synthesized by the various types of choroidal cells (endothelial, stromal and epithelial) and assessing whether translocation pathways in this tissue can relay CSF chemokines to stromal lymphocytes. The reconstituted choroidal epithelium presented in this paper will allow investigating such transcytosis mechanisms. A first set of data on CCL2 shows that the apical-to basolateral transfer of this chemokine is very low [34].

The adhesion molecules involved in the migration process have not been investigated in this study. They may also be part of CNS-specific trafficking determinants. At the choroid plexus, besides the likely involvement of endothelial P-selectin [4], the molecular partners involved in endothelial diapedesis, in further migration through the stroma, and in transepithelial trafficking, remain to be deciphered. Given the lack of shear stress imposed on stromal T cells, mechanisms alternate to those described in endothelia are likely to be involved at the blood-CSF barrier proper.

In accord with its potential implication in “routine immunosurveillance”, T cell migration through the choroidal monolayer in vitro did not induce tight junction protein loss at the epithelial borders as assessed by immunocytochemical staining of claudins. The integrity of the epithelium assessed by its permeability to sucrose was also preserved, implying that T-lymphocyte migration is a tightly regulated mechanism. Half of the T-lymphocytes adhering to the epithelium and contacting intercellular junctions were associated with multicellular corners. Given the length of bicellular junctions relative to the restricted tricellular area available at tricellular corners, this ratio reflects a preferential localization of T cells at these sites. The lack of apparent discontinuity in claudin immunoreactivity at these corners argues against the previously proposed concept that tricellular corners are regions of interrupted junctions and of least resistance, favoring cell migration [39]. A specific feature of tricellular corner junctions between epithelial cells is their enrichment into tricellulin, a tetraspan protein belonging with occludin to the tight junction associated Marvel protein family. This protein plays a critical role in tight junction permeability and function [40]. Of note, this protein concentrates in vertically oriented strands in tricellular tight junctions in mouse Eph4 epithelial cell monolayers, and tricellulin immunoreactivity clearly extends basolaterally to occludin labelling. Whether this protein or some other distinct feature of the tricellular corner mediates the preferential interaction and migration of immune cells at this location needs further investigation. ‘‘Funnel-like” structures forming from the apical membrane were observed in a porcine choroidal monolayer to allow migration of paracellularly located neutrophils blocked by the tight junction complex. [30]. Whether T cells share a similar pathway of migration remains to be investigated. Current strategies developed to treat inflammatory disorders of the CNS are primarily directed at blocking immune cell adhesion or chemokine/chemokine receptor interactions, and target the inflamed blood-brain barrier. The present data provide evidence for the controlled migration of T cells across the blood-CSF barrier into brain, and further indicate that this recruitment route is sensitive to CSF chemokines. Therapeutic strategies will therefore benefit from identifying immune cell ligands and their counter receptors involved in normal immune surveillance across the choroid plexus, defining the profile, extent, and polarity of chemokines secreted by the choroidal epithelium per se, and finally investigating how these key factors are affected during chronic neuroinflammation.

## References

[1] Ransohoff RM, Kivisakk P, Kidd G (2003) Three or more routes for leukocyte migration into the central nervous system. Nat Rev Immunol 3: 569–581. 12876559 10.1038/nri1130

[2] Ransohoff RM, Engelhardt B (2012) The anatomical and cellular basis of immune surveillance in the central nervous system. Nat Rev Immunol 12: 623–635. 10.1038/nri3265 22903150

[3] Giunti D, Borsellino G, Benelli R, Marchese M, Capello E, Valle MT, et al (2003) Phenotypic and functional analysis of T cells homing into the CSF of subjects with inflammatory diseases of the CNS. J Leukoc Biol 73: 584–590. 12714572 10.1189/jlb.1202598

[4] Kivisakk P, Mahad DJ, Callahan MK, Trebst C, Tucky B, Rudick RA, et al (2003) Human cerebrospinal fluid central memory CD4+ T cells: evidence for trafficking through choroid plexus and meninges via P-selectin. Proc Natl Acad Sci U S A 100: 8389–8394. 12829791 10.1073/pnas.1433000100 PMC166239

[5] Provencio JJ, Kivisakk P, Tucky BH, Luciano MG, Ransohoff RM (2005) Comparison of ventricular and lumbar cerebrospinal fluid T cells in non-inflammatory neurological disorder (NIND) patients. J Neuroimmunol 163: 179–184. 15885320 10.1016/j.jneuroim.2005.03.003

[6] Yang J, Galipeau J, Kozak CA, Furie BC, Furie B (1996) Mouse P-selectin glycoprotein ligand-1: molecular cloning, chromosomal localization, and expression of a functional P-selectin receptor. Blood 87: 4176–4186. 8639776

[7] Carrithers MD, Visintin I, Kang SJ, Janeway CA Jr (2000) Differential adhesion molecule requirements for immune surveillance and inflammatory recruitment. Brain 123 (Pt 6): 1092–1101. 10825349 10.1093/brain/123.6.1092

[8] Piccio L, Rossi B, Scarpini E, Laudanna C, Giagulli C, Issekutz AC, et al (2002) Molecular mechanisms involved in lymphocyte recruitment in inflamed brain microvessels: critical roles for P-selectin glycoprotein ligand-1 and heterotrimeric G(i)-linked receptors. J Immunol 168: 1940–1949. 11823530 10.4049/jimmunol.168.4.1940

[9] Petito CK, Adkins B (2005) Choroid plexus selectively accumulates T-lymphocytes in normal controls and after peripheral immune activation. J Neuroimmunol 162: 19–27. 15833356 10.1016/j.jneuroim.2004.12.020

[10] Schmitt C, Strazielle N, Ghersi-Egea JF (2012) Brain leukocyte infiltration initiated by peripheral inflammation or experimental autoimmune encephalomyelitis occurs through pathways connected to the CSF-filled compartments of the forebrain and midbrain. J Neuroinflammation 9: 187 22870891 10.1186/1742-2094-9-187 PMC3458946

[11] Reboldi A, Coisne C, Baumjohann D, Benvenuto F, Bottinelli D, Lira S, et al (2009) C-C chemokine receptor 6-regulated entry of TH-17 cells into the CNS through the choroid plexus is required for the initiation of EAE. Nat Immunol 10: 514–523. 10.1038/ni.1716 19305396

[12] Sallusto F, Impellizzieri D, Basso C, Laroni A, Uccelli A, Lanzavecchia A, et al (2012) T-cell trafficking in the central nervous system. Immunol Rev 248: 216–227. 10.1111/j.1600-065X.2012.01140.x 22725964

[13] Kelder W, McArthur JC, Nance-Sproson T, McClernon D, Griffin DE (1998) Beta-chemokines MCP-1 and RANTES are selectively increased in cerebrospinal fluid of patients with human immunodeficiency virus-associated dementia. Ann Neurol 44: 831–835. 9818943 10.1002/ana.410440521

[14] Ubogu EE, Cossoy MB, Ransohoff RM (2006) The expression and function of chemokines involved in CNS inflammation. Trends Pharmacol Sci 27: 48–55. 16310865 10.1016/j.tips.2005.11.002

[15] Brown DA, Sawchenko PE (2007) Time course and distribution of inflammatory and neurodegenerative events suggest structural bases for the pathogenesis of experimental autoimmune encephalomyelitis. J Comp Neurol 502: 236–260. 17348011 10.1002/cne.21307

[16] Allen IV, Kirk J (1997) The anatomical and molecular pathology of multiple sclerosis In: Russell WC, editor. Molecular biology of multiple sclerosis. Chichester: Wiley and sons pp. 9–22.

[17] Kutzelnigg A, Lassmann H (2005) Cortical lesions and brain atrophy in MS. J Neurol Sci 233: 55–59. 15893328 10.1016/j.jns.2005.03.027

[18] Strazielle N, Ghersi-Egea JF (2000) Choroid plexus in the central nervous system: biology and physiopathology. J Neuropathol Exp Neurol 59: 561–574. 10901227 10.1093/jnen/59.7.561

[19] Tsutsumi M, Skinner MK, Sanders-Bush E (1989) Transferrin gene expression and synthesis by cultured choroid plexus epithelial cells. Regulation by serotonin and cyclic adenosine 3',5'-monophosphate. J Biol Chem 264: 9626–9631. 2542315

[20] Foudi A, Jarrier P, Zhang Y, Wittner M, Geay JF, Lecluse Y, et al (2006) Reduced retention of radioprotective hematopoietic cells within the bone marrow microenvironment in CXCR4-/- chimeric mice. Blood 107: 2243–2251. 16291599 10.1182/blood-2005-02-0581

[21] Strazielle N, Belin MF, Ghersi-Egea JF (2003) Choroid plexus controls brain availability of anti-HIV nucleoside analogs via pharmacologically inhibitable organic anion transporters. Aids 17: 1473–1485. 12824785 10.1097/00002030-200307040-00008

[22] Strazielle N, Ghersi-Egea JF (1999) Demonstration of a coupled metabolism-efflux process at the choroid plexus as a mechanism of brain protection toward xenobiotics. J Neurosci 19: 6275–6289. 10414957 10.1523/JNEUROSCI.19-15-06275.1999 PMC6782833

[23] Siflinger-Birnboim A, Del Vecchio PJ, Cooper JA, Blumenstock FA, Shepard JM, Malik AB (1987) Molecular sieving characteristics of the cultured endothelial monolayer. J Cell Physiol 132: 111–117. 3597548 10.1002/jcp.1041320115

[24] Saito Y, Wright EM (1983) Bicarbonate transport across the frog choroid plexus and its control by cyclic nucleotides. J Physiol 336: 635–648. 6308232 10.1113/jphysiol.1983.sp014602 PMC1198989

[25] Villalobos AR, Miller DS, Renfro JL (2002) Transepithelial organic anion transport by shark choroid plexus. Am J Physiol Regul Integr Comp Physiol 282: R1308–1316. 11959670 10.1152/ajpregu.00677.2001

[26] Pappenheimer JR, Heisey SR, Jordan EF (1961) Active transport of Diodrast and phenolsulfonphtalein from cerebrospinal fluid to blood. Am J Physiol 200: 1–10. 13732693 10.1152/ajplegacy.1961.200.1.1

[27] Miller LA, Butcher EC (1998) Human airway epithelial monolayers promote selective transmigration of memory T cells: a transepithelial model of lymphocyte migration into the airways. Am J Respir Cell Mol Biol 19: 892–900. 9843923 10.1165/ajrcmb.19.6.3245mrev

[28] Parkos CA, Delp C, Arnaout MA, Madara JL (1991) Neutrophil migration across a cultured intestinal epithelium. Dependence on a CD11b/CD18-mediated event and enhanced efficiency in physiological direction. J Clin Invest 88: 1605–1612. 1682344 10.1172/JCI115473 PMC295682

[29] Tenenbaum T, Papandreou T, Gellrich D, Friedrichs U, Seibt A, Adam R, et al (2009) Polar bacterial invasion and translocation of Streptococcus suis across the blood-cerebrospinal fluid barrier in vitro. Cell Microbiol 11: 323–336. 10.1111/j.1462-5822.2008.01255.x 19046337

[30] Wewer C, Seibt A, Wolburg H, Greune L, Schmidt MA, Berger J, et al (2011) Transcellular migration of neutrophil granulocytes through the blood-cerebrospinal fluid barrier after infection with Streptococcus suis. J Neuroinflammation 8: 51 10.1186/1742-2094-8-51 21592385 PMC3120695

[31] Kunis G, Baruch K, Rosenzweig N, Kertser A, Miller O, Berkutzki T, et al (2013) IFN-gamma-dependent activation of the brain's choroid plexus for CNS immune surveillance and repair. Brain 136: 3427–3440. 10.1093/brain/awt259 24088808

[32] Schneider H, Weber CE, Schoeller J, Steinmann U, Borkowski J, Ishikawa H, et al (2012) Chemotaxis of T-cells after infection of human choroid plexus papilloma cells with Echovirus 30 in an in vitro model of the blood-cerebrospinal fluid barrier. Virus Res 170: 66–74. 10.1016/j.virusres.2012.08.019 23000117

[33] Kivisakk P, Mahad DJ, Callahan MK, Sikora K, Trebst C, Tucky B, et al (2004) Expression of CCR7 in multiple sclerosis: implications for CNS immunity. Ann Neurol 55: 627–638. 15122702 10.1002/ana.20049

[34] Szmydynger-Chodobska J, Strazielle N, Gandy JR, Keefe TH, Zink BJ, Ghersi-Egea JF, et al (2012) Posttraumatic invasion of monocytes across the blood-cerebrospinal fluid barrier. J Cereb Blood Flow Metab 32: 93–104. 10.1038/jcbfm.2011.111 21829211 PMC3323293

[35] Szmydynger-Chodobska J, Strazielle N, Zink BJ, Ghersi-Egea JF, Chodobski A (2009) The role of the choroid plexus in neutrophil invasion after traumatic brain injury. J Cereb Blood Flow Metab 29: 1503–1516. 10.1038/jcbfm.2009.71 19471279 PMC2736364

[36] Kivisakk P, Trebst C, Liu Z, Tucky BH, Sorensen TL, Rudick RA, et al (2002) T-cells in the cerebrospinal fluid express a similar repertoire of inflammatory chemokine receptors in the absence or presence of CNS inflammation: implications for CNS trafficking. Clin Exp Immunol 129: 510–518. 12197893 10.1046/j.1365-2249.2002.01947.x PMC1906480

[37] Zhang X, Wu C, Song J, Gotte M, Sorokin L (2013) Syndecan-1, a cell surface proteoglycan, negatively regulates initial leukocyte recruitment to the brain across the choroid plexus in murine experimental autoimmune encephalomyelitis. J Immunol 191: 4551–4561. 10.4049/jimmunol.1300931 24078687

[38] Mordelet E, Davies HA, Hillyer P, Romero IA, Male D (2007) Chemokine transport across human vascular endothelial cells. Endothelium 14: 7–15. 17364892 10.1080/10623320601177312

[39] Burns AR, Walker DC, Brown ES, Thurmon LT, Bowden RA, Keese CR, et al (1997) Neutrophil transendothelial migration is independent of tight junctions and occurs preferentially at tricellular corners. J Immunol 159: 2893–2903. 9300713

[40] Ikenouchi J, Furuse M, Furuse K, Sasaki H, Tsukita S, Tsukita S (2005) Tricellulin constitutes a novel barrier at tricellular contacts of epithelial cells. J Cell Biol 171: 939–945. 16365161 10.1083/jcb.200510043 PMC2171318

[41] Strazielle N, Khuth ST, Ghersi-Egea JF (2004) Detoxification systems, passive and specific transport for drugs at th blood-CSF barrier in normal and pathological situations. Adv Drug Deliv Rev 56: 1717–1740. 15381331 10.1016/j.addr.2004.07.006

